# Frame-based Programming, Stream-Based Processing for Medical Image Processing Applications

**DOI:** 10.1007/s11265-018-1422-3

**Published:** 2019-01-04

**Authors:** Joost Hoozemans, Rob de Jong, Steven van der Vlugt, Jeroen Van Straten, Uttam Kumar Elango, Zaid Al-Ars

**Affiliations:** 1 Computer Engineering Laboratory, Delft University of Technology, Delft, The Netherlands; 2 Philips Healthcare, Best, The Netherlands

**Keywords:** FPGA, Image processing, Medical imaging

## Abstract

This paper presents and evaluates an approach to deploy image and video processing pipelines that are developed frame-oriented on a hardware platform that is stream-oriented, such as an FPGA. First, this calls for a specialized streaming memory hierarchy and accompanying software framework that transparently moves image segments between stages in the image processing pipeline. Second, we use softcore VLIW processors, that are targetable by a C compiler and have hardware debugging capabilities, to evaluate and debug the software before moving to a High-Level Synthesis flow. The algorithm development phase, including debugging and optimizing on the target platform, is often a very time consuming step in the development of a new product. Our proposed platform allows both software developers and hardware designers to test iterations in a matter of seconds (compilation time) instead of hours (synthesis or circuit simulation time).

## Introduction

The goal of interventional medical imaging equipment is to provide the physician with real-time images from the anatomy of the patient while performing a medical intervention. One type of such equipment is the interventional X-ray (iXR) system. Typical interventions using the system include repairing blood vessel deformations such as aneurysms by positioning stents or replacing heart valves. During these procedures, blood vessels are filled with a contrast medium, which is visualized by X-rays and shown in real-time high resolution video images to the physician. As radiation is harmful to patients, doses need to be kept to a minimum. Using lower doses leads to more noise in the images, which can be reduced by using image processing filters.

The iXR is a complex system with strong real-time requirements. The system consists of many different compute architectures. The image processing algorithms are often closely tuned to the platform architecture. This makes it difficult to service the systems. At the same time, FPGA (SoC) platforms are interesting for these medical systems due to the long life time, strong performance and good real-time capabilities. However, FPGAs are often perceived as being difficult to design for. In order to address these issues, we have investigated enablers for portability towards FPGAs exploiting novel tools and techniques such as High Level Synthesis (HLS) tools. HLS is a promising approach, but currently still a specialistic toolflow requiring many code changes and optimization steps to achieve performance. Especially when moving from frame based video and image processing algorithms to a streaming implementation.

Image or video processing algorithm development is mainly done in a frame based manner which allows random access of the frame and parallelization techniques such as tiling. FPGA accelerators cannot buffer a full frame during processing, due to amongst others memory bandwidth, power and latency requirements. Therefore, the algorithm has to be implemented in a stream-based manner, where we wish to process pixels as soon as they come in and, as quickly as possible, pass the result on to the next processing step (accelerator). The current HLS tools require labor intensive hand optimizations such as using line buffers and data re-ordering instead of random memory access. There is a need to abstract away this implementation level in order to ease the FPGA implementation for the programmer.

Frameworks exist that facilitate mapping computations to FPGA (including frameworks specifically targeting image processing), but these do not solve the frame versus stream problem. Mapping the frame-based software to a stream-based hardware platform on FPGA creates the following challenges; creating a framework that moves and buffers data (in the form of image segments) between stages, causing the develop/test/optimize cycle time to increase tremendously because of synthesis.

**Figure 1:**
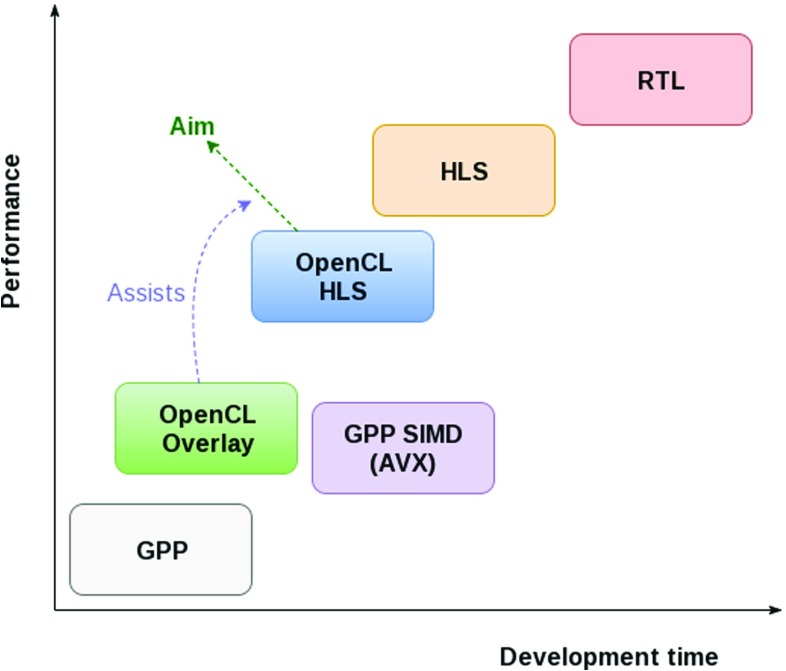
High-Level Synthesis (HLS) aims to reduce development time compared to a full-custom RTL design. Recently, FPGA vendors started to support OpenCL code. Starting from an OpenCL program, it costs less time to synthesize the first working design to FPGA, but it requires a considerable number of time-consuming test, debug, and optimization cycles before it starts to perform comparable to an HLS design. Using an FPGA overlay accelerates this process. General-Purpose Processors (GPP) have the advantage of being commodities, but improving their performance using SIMD (Single Instruction, Multiple Data) is time-consuming [1] and not portable.

In this paper, we propose an approach to solve these challenges by using an FPGA overlay fabric consisting of softcore processors that are targetable by OpenCL and a streaming memory framework. After an initial synthesis of the platform, the time required to test iterations reduces from hours (synthesis time) to seconds (compilation time). The aim is to allow the final design to achieve better performance at reduced development time (see Fig. 1).

This paper is organized as follows. Section 2 discusses the background and related work. Section 3 proposes the approach of developing frame-based programs for stream-based architectures. Section 4 presents the hardware implementation of the platform, while Section 5 presents the software stack. Section 6 discusses the experimental results performed on the platform, and Section 7 ends with the conclusions.

## Related Work

A common aim in the development of support frameworks for specific application domains is to reduce Non-Recurring Engineering costs by speeding up development time and facilitating component re-use. The performance may be slightly negatively affected (a specific full-custom design is hard to beat), but that is usually offset by lower development costs and shorter time-to-market [2].

In this section, we first discuss general approaches to speed up image processing workloads, then proceed to focus on FPGA acceleration including software support approaches, followed by a discussion of hardware support approaches using overlays and image processing fabrics and hardware integration frameworks.

### Accelerating Image Processing Workloads

Currently, there are numerous ways to either optimize image processing code or mapping it to FPGA/GPU. On common ARM and x86-based systems, Single Instruction, Multiple Data (SIMD) instruction set extensions such as AVX can be exploited to gain considerable performance [3]. There exists efforts to be able to insert these instructions automatically and some compiler support exists. When a new generation of processors or SIMD extensions is introduced, however, code must be optimized, leading to large costs in testing and validation. A study about optimizing HEVC using the AVX SIMD extension concludes that ”The large speedup, however, could only be achieved with high programming complexity and effort.” [1].

GPUs suffer from the problem of performance portability [4]. This means that for a new GPU generation, the same issue arises where engineering effort may be required to optimize the code again. Additionally, GPUs primarily target floating point calculations, that are usually algorithmically not necessary for image processing.

To facilitate the optimization process and to aid design space exploration for various target execution platforms, the Halide programming language and compiler can be used to generate code from a functional description of a filter [5]. Mapping parallel computations to a variety of computational fabrics (including multicore CPUs, GPUs and FPGA) can be done using OpenCL [6]. An overview of the challenges in designing embedded image processing systems (including a discussion of the gap between high-level algorithm description and low-level hardware design methods) is given in [7].

### FPGA Acceleration

Mapping computations to FPGA can be performed in a number of ways. High-Level Synthesis [8] is becoming a standard tool in many FPGA design environments. Additionally, FPGA vendors are supporting OpenCL through for example SDAccel [9]. The traditional approach of developing datapath designs in VHDL can still be utilized for very specific designs or if the HLS tools are not able to meet certain requirements. In the image processing application domain, the HLS toolflow has some drawbacks. Code modifications are often necessary, as it is not possible to write code in a frame-based way. Efficient tools need to be able to identify buffers in such a way that it can be mapped to FPGA efficiently (stream based) to prevent prohibitively slow main memory accesses. The ROCCC HLS compiler [10] is able to insert smart buffers that can provide some data reuse, and in [11] this concept has been extended into a framework that can generate VHDL code for sliding window filters with optimized memory structure.

Other related efforts exist, that aim to generate streaming designs from C code [12] or make use of Domain-Specific Languages (DSL), such as Halide, are Darkroom [13] and HIPAcc [14]. These approaches are able to generate hardware components for FPGA by providing an abstraction layer for HLS. Using HLS and frameworks that generate HDL code, quality of result is not always consistent and synthesis times are still very long. This means there is still a gap to be bridged between the image processing code and FPGA development.

### FPGA Overlays

To reduce compilation time and enhance portability, FPGA overlays are becoming an interesting research area. Using an overlay on FPGAs would allow software programmers to target familiar architectures, without understanding the low-level details. MARC [15] is one such project where a multi-core architecture is used as an intermediate compilation target. It consists of one control processor and multiple processors (Cores) to perform computations. The data cores are used to run OpenCL kernels and the control core is used to schedule work to the data cores. The authors conclude that using such an overlay dramatically reduces development time and bridges the gap between hardware and software programs at an acceptable performance hit compared to hand-optimized FPGA implementation. Another related effort is OpenRCL [16]. The concept of using accelerators to speed up applications while retaining programmability is discussed in [17].

In [18], a toolset is introduced for customized softcore image processing on FPGA. Customizing the softcores is a concept that can be added to our proposed framework to improve performance. Resource-efficient processing elements are introduced by [19, 20] and [21]. Our framework could make use of these processors if they were available, but our chosen processor supports OpenCL and we provide our own design-space exploration. A similar framework has been introduced in [22], but instead of providing stream-based processing that allows scalability, they employ shared memory in a banked organization, accessible via a crossbar. This reduces scalability as will be evaluated in Section 6.

### Integration Frameworks

There have been related efforts in creating FPGA development frameworks that facilitate development and integration. One example is RIFFA [23], an open source project that provides communication and synchronization between host and FPGA accelerators using PCIe. As we are using I/O directly connected to the FPGA board, we do not require PCIe interfacing. A commercial framework that can incorporate HSL-generated accelerators, hand-written VHDL and IP is the DYnamic Process LOader or Dyplo from Topic Embedded Products [24]. It incorporates a network on chip which connects both software functions and FPGA accelerators together. The network can be re-routed at run time and designated areas of the FPGA can be re-configured with a different accelerator through Xilinx Partial Reconfiguration [25].

## Approach

This section will outline how we have used a slightly modified view on the OpenCL programming model to target our proposed streaming-based hardware framework while using ordinary OpenCL kernels.

### OpenCL’s View on Parallel Computing

**Figure 2:**
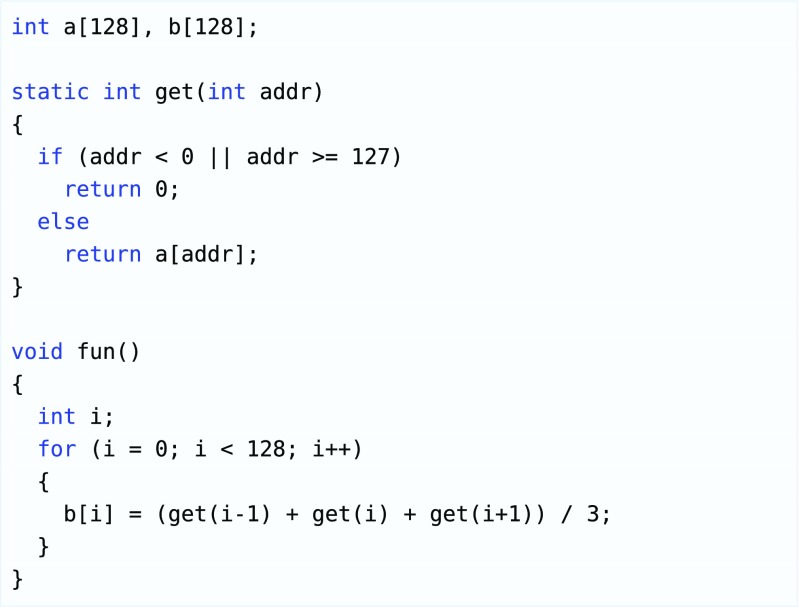
Code example of an OpenCL kernel.

In many cases a compute ‘problem’ consists of a data-set for which each element needs to undergo a certain transformation and basically this transformation is the same for each element. Consider the code example in Fig. 2: Every b[i] is produced by the exact same code fragment and there is no dependency of an element of b[] to another element of b[]. Such an operation is called a ‘kernel’ in OpenCL terminology. Conceptually, all 128 computations could have been executed concurrently, on a platform that provides 128 processing elements. This kind of parallelism is the main target of OpenCL: execution of as many kernels in parallel as possible. Note that the code for each kernel is identical, but the execution flow can be different for example due to the boundary checking ‘if’ statement in the ‘get()’ function. The OpenCL framework tries to have many accelerators performing the same operation on many datasets independently. One element of such a dataset is called a ‘work-item’.

### OpenCL Memory Model

OpenCL defines 4 types of memory objects:

1. Global Memory – read/write accessible from both the host and the execution device
2. Constant Memory – like Global Memory, but read-only for execution devices
3. Local Memory – only accessible within (a group of) execution devices
4. Private Memory – only accessible from a single execution device

**Figure 3:**
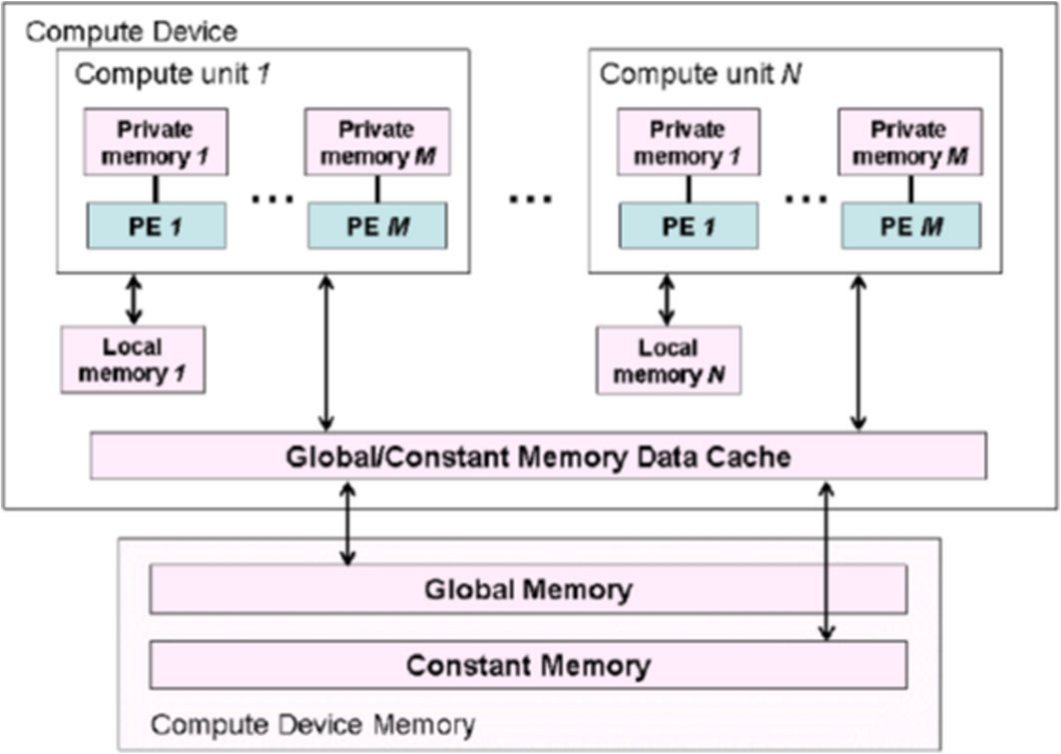
OpenCL memory structure.

OpenCL also defines a data cache between the Global / Constant memories and the execution devices. This cache is optional, but in practice it is always needed to avoid slow-down due to data transfers. This cache needs to be carefully designed, as many cores will try to access it simultaneously and in case the cache does not have enough access ports that immediately creates a new bottleneck. Note that Global / Constant memory can be physically located either on the host side or on the compute devices. When located at the host side, the compute devices need a pull mechanism to retrieve the argument data (work-item) which is inherently slower than the push mechanism the host uses when the memory is located on the devices (and conversely it is faster for results). Also note that this model uses a frame-buffer approach, not a streaming approach. Figure 3 shows the OpenCL memory structure.

### Streaming Data and OpenCL

**Figure 4:**
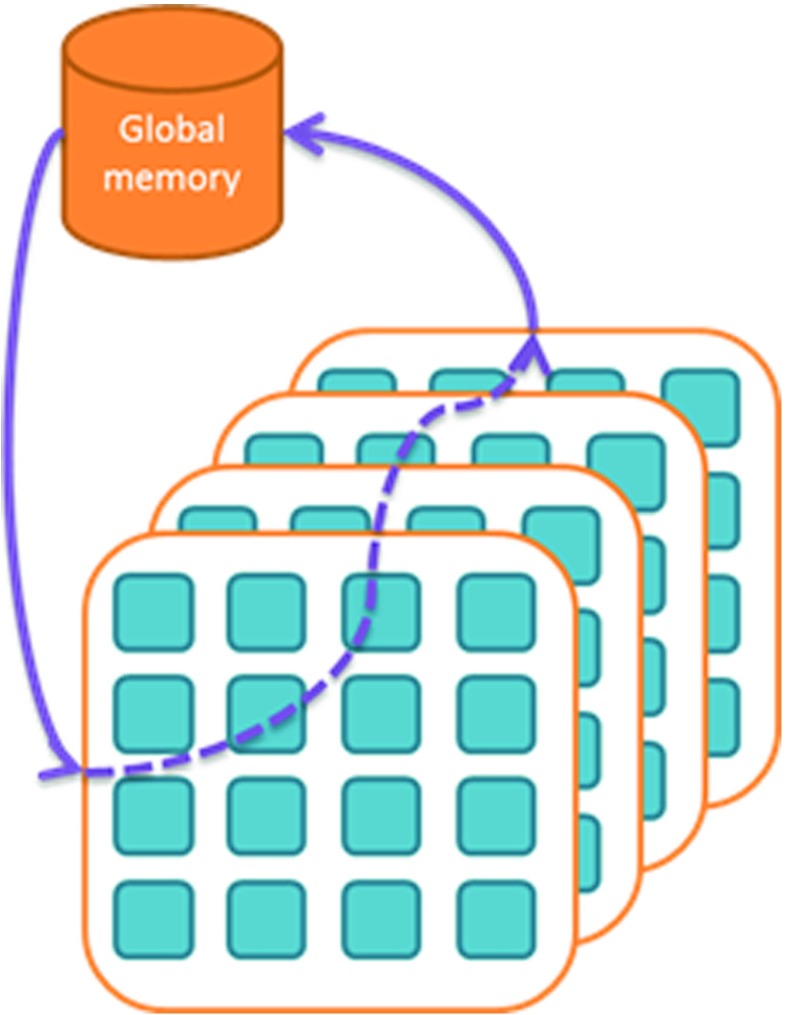
OpenCL data model.

**Figure 5:**
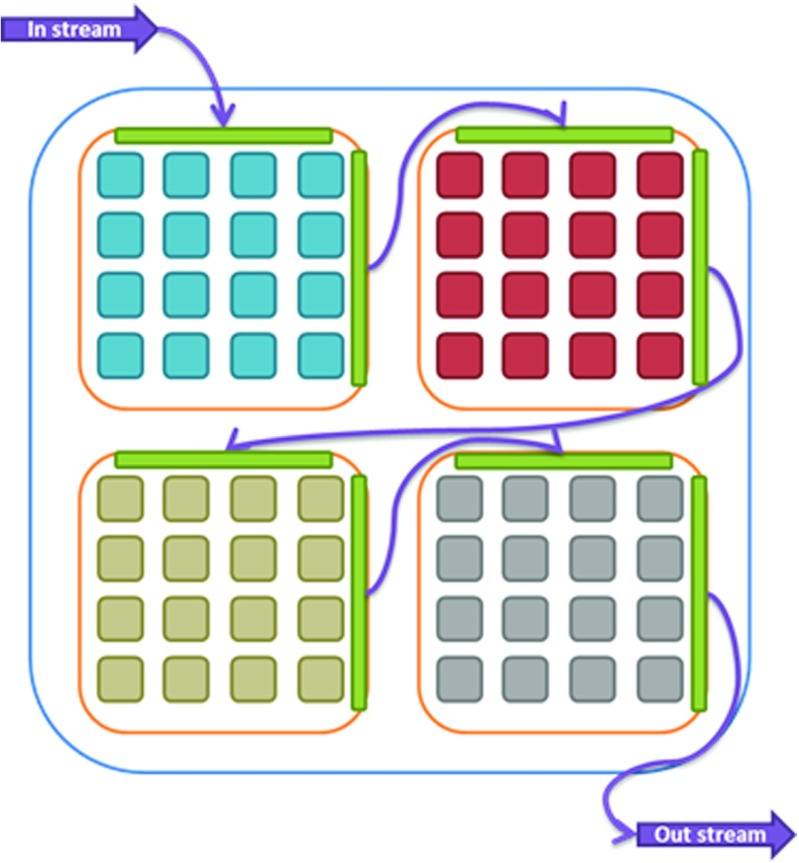
Streaming data model.

The OpenCL model works with Single Instruction Multiple Data (SIMD) processing. One set of kernels is operating on the full data set. Other sets of kernels have to be programmed each time for iterative processing, where the data is stored to the global memory in between processing steps. This approach is shown in Fig. 4. We would like to use OpenCL in a data pipelined, or streaming, implementation as explained in the previous section. This means that we would like a situation where we can program different sets of kernels where data is passed on from one set to the next, as depicted in Fig. 5. Here, results are no longer written back to global memory, but passed to other accelerators through a connection mechanism that needs to be scalable (as we are targeting highly parallel workloads running on large numbers of compute devices) and able to provide sufficient bandwidth. This can be a Network on Chip or a certain connection topology that suits the application.

Note that an alternative approach could be to execute the different kernels consecutively on every compute device (essentially, we are changing the kernel instead of moving the data - keeping the data set local). This requires every compute device to be capable of storing the instruction stream of each kernel in local instruction memory or cache. The storage capacity of these memories is an important design parameter as is explored in Section 4.1. Additionally, the size of the instruction stream will typically be larger than the size of a data block.

### OpenCL Data Architecture

**Figure 6:**
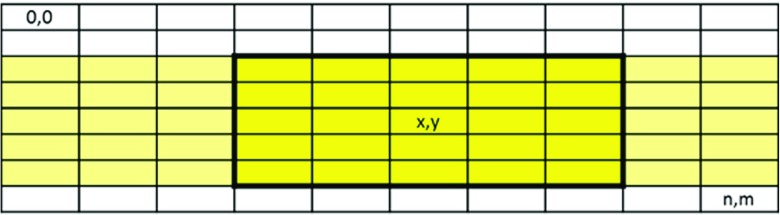
A kernel only needs a limited view on the total working set.

In OpenCL a kernel always has a full view on the entire dataset but in most cases that is not necessary. Given that a certain kernel is operating on a block of data, this kernel only needs a limited view on the total working set as depicted in Fig. 6. In this example (a 5x5 convolution kernel), the data located more than 2 lines above the current coordinates (x,y) are not needed anymore and the lines more than 2 lines below (x,y) are not needed yet. Assuming a set of compute devices are processing the data line by line, each device requires 5 lines of storage capacity to store their working set. A control mechanism should feed each compute device with the appropriate data in time and keep track of the locations of lines when assigning tasks to ensure the needed input lines are present and output results are only overwriting stale data. The OpenCL system provides a suitable basis to build such a mechanism: the command queue. This queue distributes work-items to the compute units, so it knows exactly which work-items are being processed. Consequently, it knows about the maximum view of each kernel and can compute the required data (sub)set as well.

Determining the maximum view size of the kernels in the image processing pipeline also influences certain parameters of the required hardware infrastructure (the storage capacity of the local memories of the compute devices). Conversely: for a given size of hardware buffers there is a maximum view size for each kernel. The process of finding the optimal buffer sizes between all kernels is important to prevent bottlenecks while minimizing the required sizes. There are numerous approaches to solve this, for example by performing simulations with iteratively decreasing buffer sizes, but this is outside the scope of this work. In our reference platform, we assume all filters have a maximum window size of 5x5 and will therefore need a buffer size of 5 lines for input and 1 additional line for output. The width of the lines depends on the stripe length (e.g., how the image is divided into vertical stripes), which also determines how many vertical lines of pixels need to be processed redundantly.

## Implementation - Hardware

An FPGA-based platform targeting image processing pipelines needs a number of elements; a streaming memory structure, processing units, one or more DMA units, interfaces with off-board electronics (to receive the image and output it after processing), and control & debug interfaces with a central host. Additionally, run-time support is needed to move the image segments through the streaming memory structure. This should be done as transparently as possible in order to keep frame-based programmability.

### Processing Element

The processing elements used in this work are based on the *ρ*-VEX VLIW processor developed by TU Delft [26]. The implementation of this processor is written in a very generic way, so design space exploration can be performed. In our application domain, there is ample parallelism on both instruction level and data level (that can be exploited by SIMD or multithreading). The design-time configuration options available for the *ρ*-VEX are listed in Table 1.

**Table 1:** Design-time configuration options of the *ρ*-VEX processor and their effect on various exploration metrics.

Configuration	Area	Code	
option	utilization	performance	Timing
Issue-width	−−	+	−
Forwarding	−	+	−
Traps	+/−	+/−	+/−
Breakpoints	+/−	+/−	−
Perf. counters	−	+/−	−
Additional			
pipeline stages	+/−	−	+

The processor will be configured in the smallest issue width to improve timing and limit area utilization as much as possible. Any decrease in area utilization may results in a larger number of cores, which will directly improve performance. Disabling forwarding in the pipeline and adding additional pipeline stages will impact code performance due to additional latency between operations, but this penalty can be reduced or even removed by using loop unrolling in the *ρ*-VEX compiler in order to fill the latency slots with other operations. This requires the cores to have sufficiently sized instruction memories, resulting in another trade-off as the memory sizes will impact timing and, to a certain extent, the number of cores that will fit on the FPGA (Table 2).

**Table 2:** Example of the design-space exploration of the *ρ*-VEX pipeline organization, area utilization and timing.

Pipeline		N	Resource utilization			Freq.
Forward	Stgs		LUT	FF	BRAM	(MHz)
Enabled	7	64	99*%*	29*%*	81*%*	149
Enabled	5	64	93*%*	26*%*	81*%*	103
Disabled	7	75	96*%*	33*%*	95*%*	162
Disabled	5	75	98*%*	30*%*	95*%*	143
Disabled	7	4	5*%*	2*%*	5*%*	200
Disabled	7	64	82*%*	28*%*	81*%*	193

N denotes the number of cores in the design. The bottom two rows represent designs that were placed & routed using manually created placement constraints to improve timing, the upper rows are results from running without constraints.

### Memory Structure

The memory structure as used in our overlay is introduced in [27]. The concept is to organize the cores into streams of a configurable number of cores. Within such a stream, each processor has a local (scratchpad) instruction memory and a local data memory. The sizes of these memories must be set at design-time and determine the maximum size of the program (.text section of the binary), and the maximum size and number of line buffers that cores can store. Similar to the design-space exploration of the instruction memory size, as discussed in Section 4.1, the size of the data memory buffers is an important parameter that should be carefully considered. Too large data memories can limit the number of cores that can be placed on the FPGA and create timing difficulties, too small memories can results in bottlenecks in the stream or prevent a certain core from supporting certain filters (for example, a convolution filter with a 5x5 pixel window size needs at least 5 buffered input lines and a buffer to write the output line).

**Figure 7:**
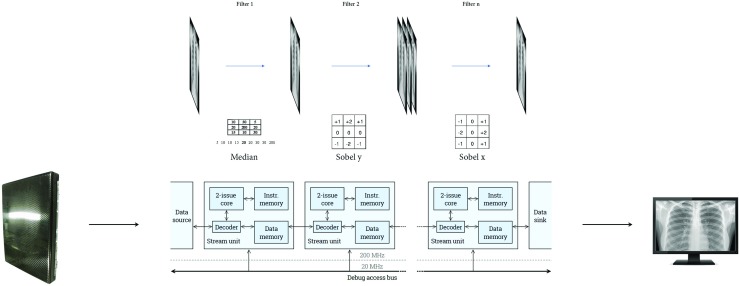
Overview of the streaming memory framework. Obtaining the image segment from the source and writing it to the sink is performed by a DMA unit that accesses framebuffers in the DRAM on the FPGA. Transfers between stream units are performed by local buses in the FPGA fabric, connecting local memories instantiated using BRAMs.

In addition to its own local data memory, each core in a stream is able to access the data memory of its *predecessor* by means of an address decoder (see Fig. 7). Each core in the stream will run a filter in the image processing pipeline. The local data memories are implemented using dual-ported BRAMs so that each core has single-cycle access to both memory regions. The first and last memories in the streams are connected to an AXI bus using a DMA unit. The image is segmented to distribute the workload over the available streams, while taking into consideration the necessary overlap to perform the window-based operations. Sending data, commands, parameters and synchronization is performed using the local memories (for example, convolution kernel parameters are propagated through the stream). Loading the instruction memories, debugging and resetting the individual cores is performed using a separate debug bus that is operating on a lower frequency to avoid timing difficulties. These last tasks are only performed during startup or debugging, therefore the lower frequency will not interfere with the performance.

### Interfaces

This section will discuss the hardware interfaces that move data to and from the processing elements and the outside world.

#### DMA Unit

**Figure 8:**
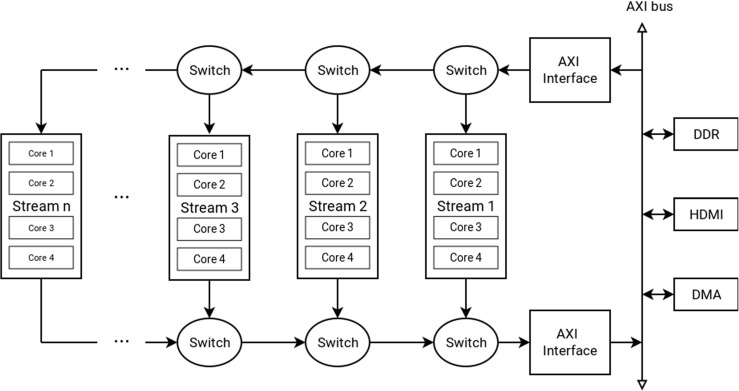
DMA unit that is in essence a simple NoC with switches leading up to an AXI bridge that bursts packets of streaming data to a framebuffer in DDR (or any memory address provided by the requesting stream).

As stated, the first and last core of each stream is connected to a DMA unit that can transfer blocks of data to the AXI bus. This connection is implemented in a non-blocking way, to allow cores to send a request to the DMA unit without having to wait until it becomes available. Arbitration is performed by means of a simplified Network-on-Chip (NoC), as is depicted in Fig. 8.

The requesting core writes the target address and the data (or size) to be transfered into a BRAM, and flags the request in a control register. Each pair of streams is connected to an arbiter (router) in a fully unbalanced organization (this creates a better layout for Place and Route). Each arbiter contains registers in order to avoid a long timing path. When receiving a new request, an arbiter will lock itself until the payload has been fully transferred (similar to wormhole switching). This way, all payloads will be transferred undivided, so the burst mode of the AXI bus can be used most effectively. The priorities of the arbiters are set such that streams that are further away from the bus interface have precedence.

#### Debug Bus

In order to support the extensive debugging capabilities that are offered by the *ρ*-VEX processor, a separate bus is created that operates on a lower frequency than the datapaths and memories. This is because this bus is not performance critical and the lower clock will facilitate timing on the FPGA. The bus is bridged to the AXI main bus by means of an AXI slave interface, and connected to all cores in the system (all of which are memory-mapped into the AXI slave’s address space). The functionality of the debug bus is determined by the memory regions of each *ρ*-VEX core that it is able to access - the instruction memory, data memory, and control registers. The set of control registers allow standard operations such as halting and resetting the core, but also more advanced requests such as register file access, setting watch/breakpoints and toggling single-step execution mode.

To be able to use all debugging functionality, the *ρ*-VEX must be configured with traps enabled. Whenever a trap occurs during execution, the core will store the cause, the location in the program (program counter value), and an argument in a control register. This way, it is possible to ascertain what went wrong before the core halted or trapped into the trap handler. For example, if the core performs a memory read to an invalid address (unmapped or unaligned address), it will show the cause associated with invalid data access, along with the program counter that contained the corresponding load instruction and the address it was trying to access.

## Implementation - Software

This section describes the implementation from a software point of view, starting with the buffer management and how the workload is parallelized over multiple cores, how the system performs the necessary synchronization, the way that OpenCL support has been implemented, how the interfaces are programmed and lastly how to develop applications for the platform.

### Compilation and Operation

The process elements, as discussed in Section 4.1, are based on the VEX instruction set architecture [28]. There is a full toolchain available for these cores, that must be used to compile code for the platform. A C compiler is available, along with a port of binutils and an architectural simulator that can be used for initial debugging of the code during development. The process of writing parallel code for the platform will be discussed in more detail in Section 5.4.

To load the binaries into all the processing elements, the current platform implementation includes a management core. In principle, this can be a (hard) ARM-based device (in case of Zynq and comparable platforms), or even an additional *ρ*-VEX core, but in our current implementation it is a microblaze processor as it is used in Xilinx reference designs to configure the AXI-based platform and peripherals. This core is not running any filters, but only concerns itself with sending commands to all the processing elements. It is able to access all the processing elements’ control registers, instruction memory and data memory by means of the debug bus. In addition, it can control the DMA unit using control registers.

At startup time, the management cores resets and halts all processing elements and loads the corresponding instruction stream into the instruction memory of each core in each stream. Then it populates a datastructure in the data memories of each core in order to have them wait for instructions (see also Section 5.3) and releases the cores. During normal operation, the instruction memories only need to be loaded once, however, it is possible to change the image processing pipeline by simply halting the cores and uploading a different instruction stream.

### Buffer Management

**Figure 9:**
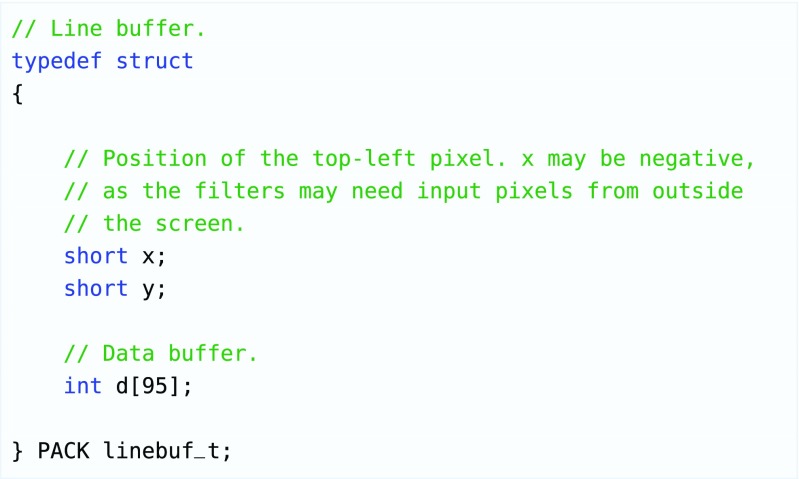
Struct that contains a single line of a stripe of the image that is being processed. It contains the position in the frame so it can be used as input for subsequent filters or written into a frame buffer.

**Figure 10:**
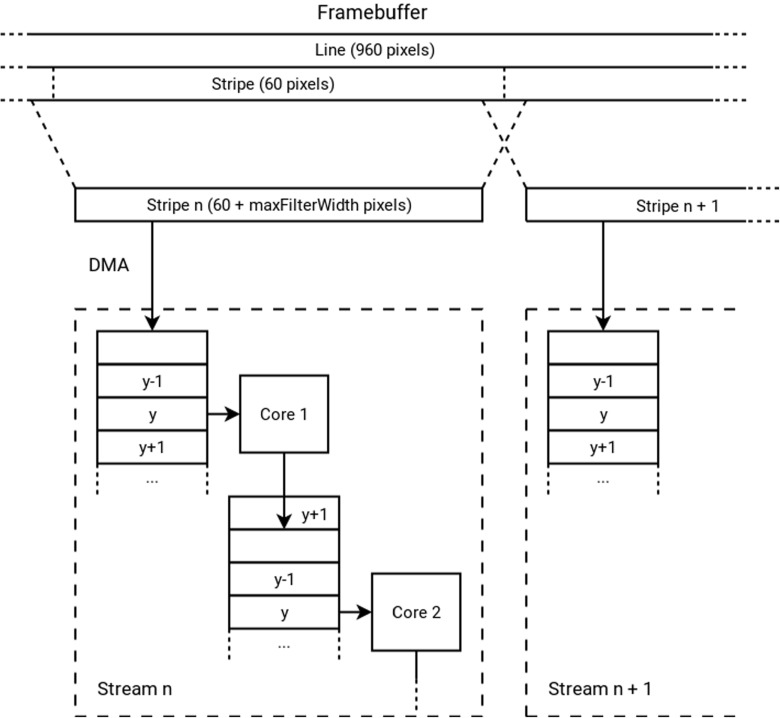
Diagram showing the overlap between stripes allocated to neighboring Streams, and lines that automatically overlap because they are allocated to the same stream.

As discussed in Section 4.2, the image processing workload will be distributed across the available cores in two dimensions - each core in a stream will perform a separate filter (task-level parallelism), and each stream will handle a part of the image (data-level parallelism). The image is divided by vertical stripes of a certain width. In our reference design, the total number of cores is 16 streams of 4 cores each and the default stripe width is 60 pixels. The stripes are buffered per line in the local data memories of the processing elements. The data structure of these buffers is depicted in Fig. 9. The type of filters that are to be supported by a certain core in the stream determines the number of line buffers that need to fit into the input data memory. If the filter operates on a 5x5 window to produce 1 output line, there must be enough storage capacity to store 5 lines in the input memory and 1 line in the output memory. Note that these storage requirements overlap between two adjacent cores. The buffers circulate in such a way that the required input lines are stable, and the line that is not needed anymore will be used as output storage for the previous core (see Fig. 10). The horizontal overlap is handled by extending the stripes on both sides when reading in the input from the framebuffer using DMA. This overlap is dependent on the filter size in the same way as the required number of buffered lines.

When distributing block-based filters among multiple streams, some amount of overlap is required between the stripes. Instead of communicating these pixels between cores (which is not supported by the memory structure), our platform redundantly computes them in every stream. The largest filter size determines the necessary amount of overlapping pixels, and these are added to the workload automatically by the management core. Edges can be dealt with in different ways; each core could implement the edge behavior and perform a check on each input line to identify whether it is an edge line. However, this will increase code size. Another option is to implement the behavior on a dedicated core and instruct the management core to send all edge lines to it. If the management core is fast enough, it could also perform the filters on all the edge lines directly.

### Synchronization and Communication

**Figure 11:**
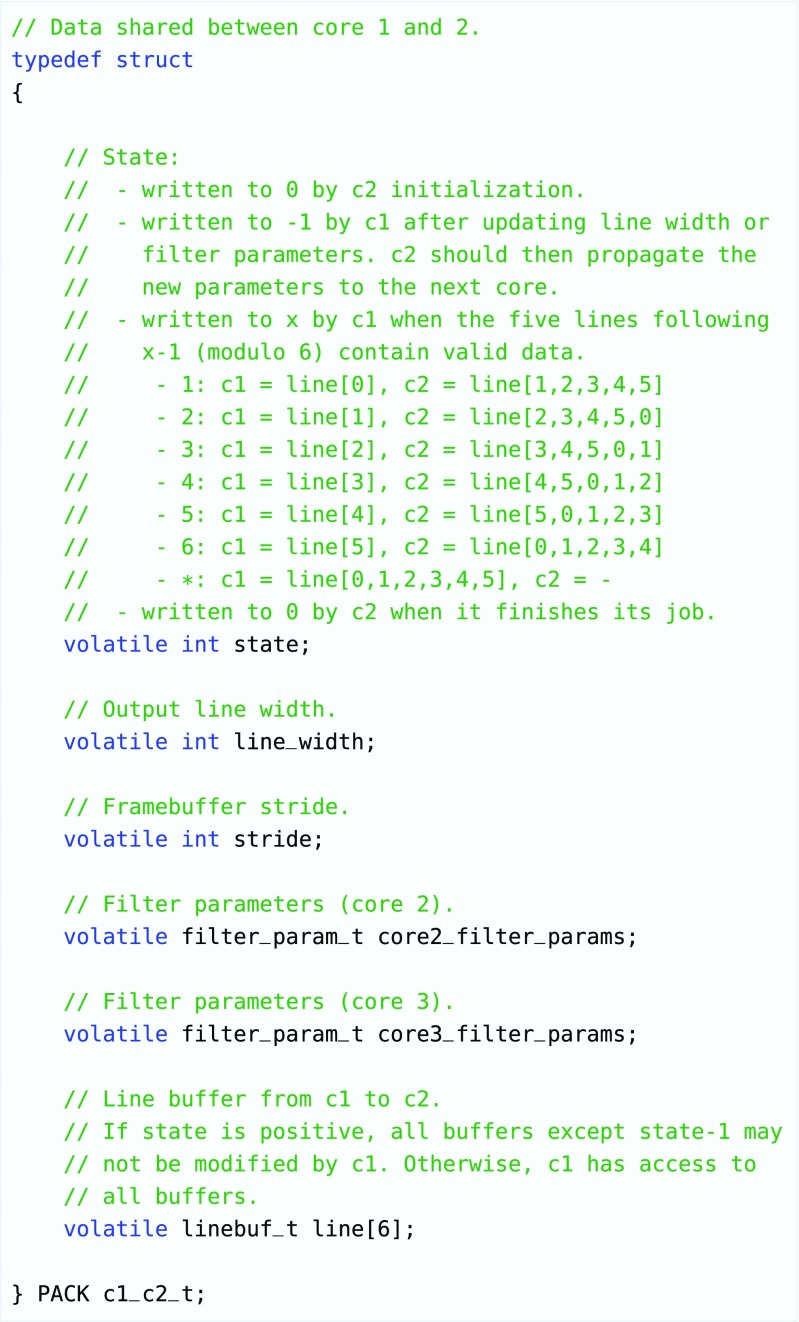
Example of a struct that is used to communicate between the cores, and to synchronize the streams. It can be used to change which filter should be performed, update filter parameters, and to apply backpressure to predecessor cores (using the *state* member). This struct is used to communicate between core 1 and 2 (therefore it does not contain any parameters for cores 0 and 1) and contains 6 linebuffers.

As the processing elements’ data memories are not connected to the AXI bus directly, but to a slower debug bus, controlling each individual core from the microblaze using this debug bus would be too slow. However, each processing element is able to receive commands from the management core by means of a data structure that is depicted in Fig. 11.

The struct contains a *state* member that is used for synchronization and to control buffer ownership. The predecessor core can take ownership of a line buffer by writing the index of that particular buffer into the state field. The buffers contain consecutive lines in a circular fashion and it is always implied that the buffers that are not reserved by the predecessor core contain valid lines (so that these can be used as input by the successor core). The successor core resets the state value to 0 when it is finished processing its line. The predecessor core will wait for this event before proceeding with the next line, so this mechanism can be used to apply backpressure.

**Figure 12:**
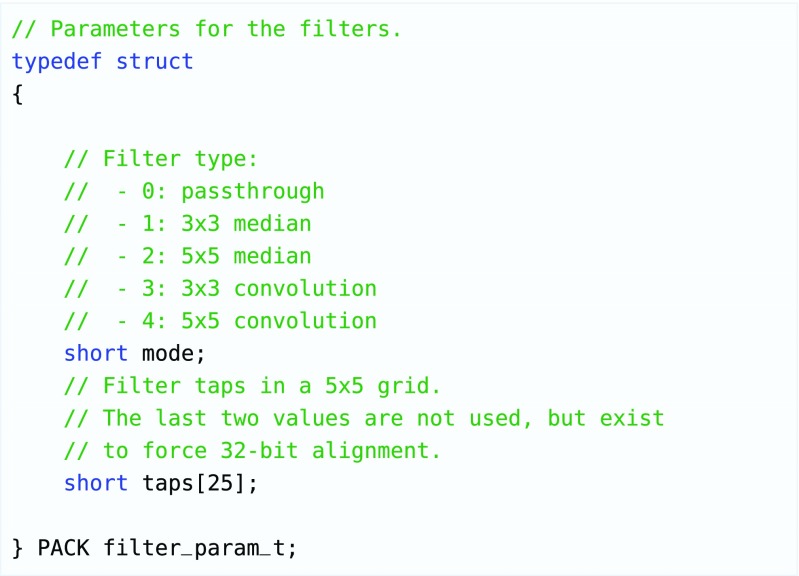
Example of a struct that is used to communicate filter parameters between cores. In theory, each core in the stream could have a specific struct because they can all have a distinct instruction memory containing different filters. In our reference implementation, the cores all support two different filters (median and convolution), and the kernel that is used for the convolution can be modified using the struct.

In addition to the state member, the structs contain a filter parameter struct for each following core. An example of this struct is depicted in Fig. 12. If the state field is set to -1, a core will propagate these values to its successor. This way, new values can be loaded into each core. Lastly, the communication struct contains a number of line buffers. As discussed, the exact number must be set by the designer after careful consideration of the filters that a certain core needs to be able to perform. Automating this using a dataflow buffer sizing analysis method could be considered as possible future work.

### Application Development

One of the related work in terms of software development for image processing filters is Halide [5]. It can generate code by describing the relationship between an output pixel and its required input pixels. This is possible because the operations are the same for each pixel, using different input pixels. As discussed in Section 3, a work-item size of a single pixel is bad because of overhead (not only on a software level, but this would also prevent the DMA engine from achieving high bus throughput as this requires burst transfers). Therefore, the platform operates on lines instead of pixels. Developing filters for the platform consists of modifying the frame-based reference implementation into a line-based implementation. In the halide analogy, instead of describing how to calculate a single pixel, the programmer must specify how to compute a line of a given length. The difficult facets of the code transformation are handled by the hardware framework as outlined in the previous sections. These include time-consuming and error-prone elements such as buffer management, dividing the workload over multiple processing elements (whose number will most likely change during the exploration phase), synchronization, and I/O (using DMA transfers).

The framework supplies the input linebuffers and an output linebuffer, the width of the line, and parameters for the filter (if applicable). Kernels can be programmed in OpenCL and compiled with our LLVM backend from the Portable OpenCL (pocl) framework [29]. Alternatively, kernels can be programmed in C or VEX assembly code. The kernel code must be linked together with a control loop that polls for work (supplied by the framework).

## Experiments/Evaluation

**Figure 13:**
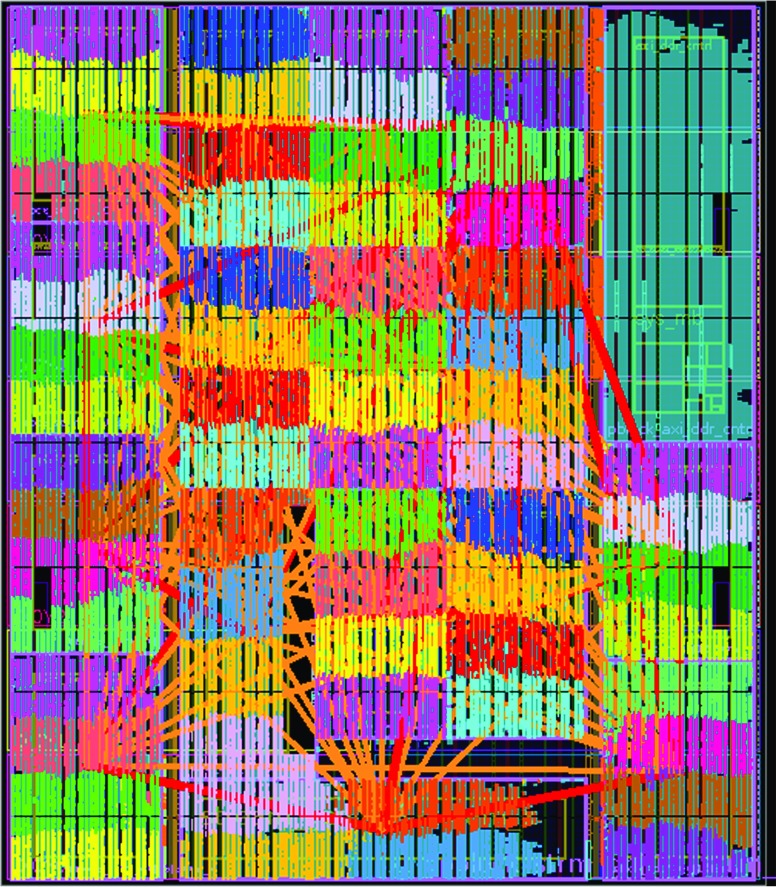
Layout of a 64-core platform on a Xilinx VC707 evaluation board. Each color represents a *ρ*-VEX 2-issue VLIW processing element. The cores are organized in 16 streams of 4 cores. The upper right corner contains additional logic such as the HDMI interface, DDR controller, Microblaze and DMA unit.

To evaluate the approach, we have developed a reference implementation using the framework and synthesized this for the Xilinx VC707 evaluation board with a Virtex 7 FPGA. The VHDL design is fully parameterized, and the fabric is organized in 16 streams of 4 consecutive cores with each 4 KiB of instruction and data memory. Figure 13 depicts the layout of the placed and routed design on the FPGA, after providing placement constraints for each individual stream of 4 cores while constraining the management core (microblaze) and interface logic such as HDMI and DDR controllers to the upper right corner.

**Figure 14:**
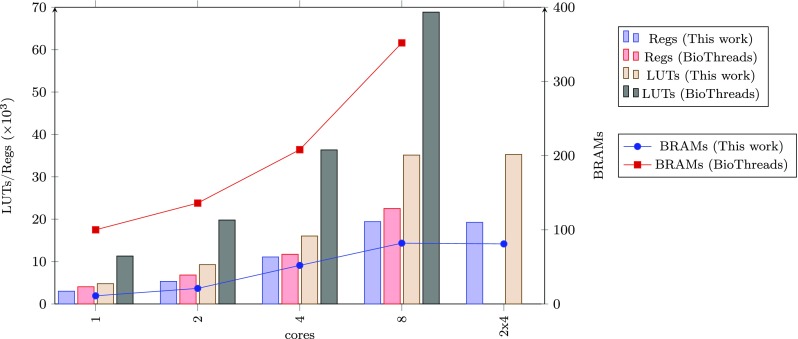
Resource utilization comparison with a related platform [22]. The advantage of this work is that it provides the option of adding additional processing pipelines instead of increasing the number of cores connected to a central memory component. This is far more scalable and allows us to place a considerably larger number of cores on an FPGA.

In Fig. 14, a comparison has been made with a related effort named BioThreads [22], showing the advantage of our memory structure regarding scalability.

The workload of the medical imaging platform consist of window-based image processing algorithms. For our evaluation, we have implemented the convolution operator that can be used with various filter kernels (which can be programmed into the processing elements as discussed in Section 5.3). The detector provides images with a resolution of 960 by 960 pixels. As our reference platform has 16 parallel streams, this results in a line size of 60 pixels. Table 3 depicts the performance of the platform for a convolution kernel using a 3x3 and 5x5 filter size.

**Table 3:** Performance for different filters of a single processing element per line, per image segment, and the total throughput assuming a 16-stream platform running at 200 MHz assuming no I/O stalls.

Algorithm	Cycles/line	Cycles/img	Frames/s
	(60 pixels)	(960x960)	(200 MHz)
Convolution 3x3	4005	3844800	52
Convolution 5x5	10216	9807360	20

## Conclusions

This paper presented our approach of using a VLIW-based FPGA fabric that allows image processing kernels to be programmed in a frame-based fashion and processed efficiently in a stream-based fashion. The hardware framework handles most of the required effort in the required code transformation by automatically assigning image segments to parallel pipelines of processing elements. Our memory structure is scalable and allows pipelines of different size with different filters to be mapped to the fabric. Instead of repeatedly synthesizing the platform, designers can explore and debug the design using this framework by only recompiling OpenCL or C code. If the platform does not provide enough throughput to satisfy the performance requirements, the code that is mapped onto the VLIW softcore processors can be passed through a HLS toolflow to produce a faster system that uses the same streaming memory structure. Results show that the platform is more scalable compared to a related image processing framework.
